# Validation of retail food outlet data from a Danish government inspection database

**DOI:** 10.1186/s12937-022-00809-6

**Published:** 2022-09-27

**Authors:** Kamille Almer Bernsdorf, Henrik Bøggild, Mette Aadahl, Ulla Toft

**Affiliations:** 1 Center for Clinical Research and Prevention, Section for Health Promotion and Prevention, Bispebjerg and Frederiksberg Hospital, Copenhagen, Denmark; 2 Epidemiology and Biostatistics, Public Health and Epidemiology Group, Aalborg University Hospital, Aalborg, Denmark; 3 Institute of Clinical Medicine, Faculty of Health and Medical Sciences, University of Copenhagen, Copenhagen, Denmark

**Keywords:** Foodscape, Retail food environment, Validity, Administrative food retail data, Ground-truthing, Sensitivity, Positive predictive value

## Abstract

**Background:**

Globally, unhealthy diet is one of the leading global risks to health, thus it is central to consider aspects of the food environment that are modifiable and may enable healthy eating. Food retail data can be used to present and facilitate analyses of food environments that in turn may direct strategies towards improving dietary patterns among populations. Though food retail data are available in many countries, their completeness and accuracy differ.

**Methods:**

We applied a systematically name-based procedure combined with a manual procedure on Danish administrative food retailer data (i.e. the Smiley register) to identify, locate and classify food outlets. Food outlets were classified into the most commonly used classifications (i.e. fast food, restaurants, convenience stores, supermarkets, fruit and vegetable stores and miscellaneous) each divided into three commonly used definitions; narrow, moderate and broad. Classifications were based on branch code, name, and/or information on the internal and external appearance of the food outlet. From ground-truthing we validated the information in the register for its sensitivity and positive predictive value.

**Results:**

In 361 randomly selected areas of the Capital region of Denmark we identified a total of 1887 food outlets compared with 1861 identified in the register. We obtained a sensitivity of 0.75 and a positive predictive value of 0.76. Across classifications, the positive predictive values varied with highest values for the moderate and broad definitions of fast food, convenience stores and supermarkets (ranging from 0.89 to 0.97).

**Conclusion:**

Information from the Smiley Register is considered to be representative to the Danish food environment and may be used for future research.

**Supplementary Information:**

The online version contains supplementary material available at 10.1186/s12937-022-00809-6.

## Background

The type of food outlet and how easy it is accessed by the population can potentially influence dietary habits. Globally, unhealthy diet is one of the leading global risks to health [1] and in Denmark just 18% of the adult population eats according to the national recommendations [2]. Numerous studies suggest that environments in which individuals and families make food-related purchases are associated with their food and beverage consumption behaviors [3–5]. Thus, it is central to consider aspects of the food environment that are modifiable and may enable healthy eating.

The foodscape, i.e., the distribution of food outlets across a determined geographical area, is one aspect of the food environment. Access to, number and the type of food outlet within a neighborhood have been associated with eating habits [6–8]. Such research often involves large-scale population studies. Accordingly, most of these studies are based on secondary source data (i.e. business data, commercial business lists or government inspections databases) due to their relatively accessible nature rather than the costly and time-consuming nature of gathering primary data. Generally, inconsistency in available and accurate data on food outlets from secondary data is an existing problem within the food environment research [9]. Further, discrepancies in study settings with respect to methodological choices, exposures (e.g. food outlet classifications) and outcomes (e.g. diet or diet-related health) have caused challenges to the interpretation of studies considering the association between the foodscape and consumption behaviors or health related outcomes [7, 9, 10].

In Denmark, retail food outlet data are freely available through a government inspection database [11], but the quality is understudied. In a previous study, Toft et al. [12] validated data on fast food outlets obtained from this database (named the Smiley Register) against the gold standard of ground-truthing (i.e. field auditing). The data were found to be relatively accurate for identifying and locating fast food outlets. Nevertheless, fast food outlets form only a small part of the overall foodscape. Thus, we examined the potential of applying a systematically name-based procedure combined with a manual procedure to the Smiley Register to identify, locate and classify many different types of registered food outlets in comparison with validation in the field.

## Methods

### Study area

The geographical area of interest in the present study involved the Capital region (excluding the island of Bornholm). In defining the specific study areas, a map of the Capital region of Denmark was imported as a layer in QGIS and geographically divided into grid cells of 250x250m (*N* = 59,060 grids). A dataset from the Smiley Register year 2017 that was classified by food outlet type beforehand (as described later on) was joined with the map to provide the best guess of prevalence on each type of food outlet. Based on this “expected prevalence” we randomly selected a number of grids for ground-truthing from the two following criteria; i) a grid should contain at least one type of food outlet ii) the final number of grids should contain 10% of each food outlet type classified in Table 1. The later proportion was set with an expectation of achieving a fair representation of the foodscape to adequately evaluate the number, type, and location of food outlets. Consequently, 336 grids were selected; of these 3 were mistakenly placed outside The Capital Region, while 4 grids were placed at an amusement park and thus inaccessible to the greater public. These were discarded leaving 329 grids. A newer dataset from the Smiley Register (February 2021) was applied to identify grids that we did not expect to include food outlets. From a freely available topographic map DAGI [13] 32 grids were selected within densely populated areas including streets, houses and other buildings, but excluding areas with forest, lakes, cemeteries and sports facilities. The purpose of these “empty” grids was to determine the proportion of true negative cells that were correctly identified as not presenting food outlets. Consequently, a total of 361 grids were selected for ground-truthing during year 2020–2021 (Additional file 1).

**Table 1:** Food outlet classifications

Outlet type	Definitions		
	Narrow	Moderate	Broad
**Fast food**	Major chain outlets only (i.e. McDonalds, Sunset boulevard, Burger King, KFC, Subway, Dominos, Max burger)	“Narrow” fast food outlets plus non-chain traditional fast food AND other fast food takeaways with no/limited seating and counter service/no waitress service	‘Moderate’ outlets, plus retail bakeries (small and chains) and chain coffee shops
**Convenience**	Petrol station stores and small kiosks (i.e. Shell, Circle K, Uno X, OK plus, Q8, Kiosks)	Narrow’ outlets, plus convenience chains selling a wider, but still limited range of fresh fruits and vegetables and frozen goods (i.e. 7-eleven, Nærkøb, Letkøb, Elite købmand).	‘Moderate’ outlets, plus grocery store chains (i.e. DagligBrugsen, LokalBrugsen, Spar, Kwikspar, Brugsen, Min købmand).
**Supermarkets**	Large chain supermarkets only. Often have long opening hours (e.g. 6 am - 11 pm or 24 h) and a wide range of facilities e.g. clothes/homeware departments (i.e. Bilka, Kvickly, SuperBrugsen, Irma, Føtex, SuperBest, SuperSpar, EuroSpar, Løvbjerg, ABC lavpris, MENY)	‘Narrow’ outlets, plus medium supermarkets with shorter opening hours and less extensive range of products and facilities (Discount supermarkets i.e. Fakta, Netto, Kiwi, Rema1000, Aldi)	‘Moderate’ outlets plus small grocery stores, as defined for ‘Convenience – broad’ i.e. Petrol station stores (Shell, Circle K, Uno X, OK plus, Q8), and convenience chains selling a wider, but still limited range of fresh fruits and vegetables and frozen goods (i.e.7-eleven, Nærkøb, Letkøb, Elite købmand, Shop n Play, Kiosks), and grocery stores (i.e. DagligBrugsen, LokalBrugsen, Spar, Kwikspar, Brugsen, Min købmand)
**Restaurants**	‘Traditional restaurants’ - restaurants serving primarily lunch/dinner meals and providing waited table service	‘Narrow’ outlets, plus restaurants serving readily prepared meals and snacks all day or with a buffet/brunch and providing waited table service (i.e. Café’s)	‘Moderate’ outlets restaurants plus coffeeshops and bakery chains (i.e. Bodenhoffs bageri, Emmerys, Lagkagehuset, Reinh van haun, Farumhus, Meyers bageri, Laglace, City bakery, Grannys house)
**Fruit and Vegetable**	Outlets are characterized by primarily retailing fruits and vegetables but typically also long-life products but with a limited supply compared with supermarkets. Non-chain convenience stores are included. Farm shops are excluded.		
**Miscellaneous**	Bakeries, Butchers and Fish mongers		

### Identification and location of food outlets administrative data

We applied data from the national food safety and hygiene regulation register (i.e. the Smiley Register) administered by The Danish Veterinary and Food Administration in the Ministry of Environment and Food (DVFA) [11]. All food business operators must be registered or approved by the DVFA. From inspections the DVFA assess how well these comply with the food regulations. The number of standard inspections vary from four per year (typical frequency for butchers, fish retailers and dairies) to as when needed. If a food outlet has good compliance and gets “elite status” the frequency may be reduced to inspection every second year. The Smiley Register is publicly available and updated daily. Thus, for each day of ground-truthing we downloaded data from the Smiley Register to evaluate food outlets found in the grids against current food outlet data. Information on food outlets (e.g. postal code, outlet name, branch code, geocoordinates as decimal degrees) were extracted from the datasets. As the register include outlets, restaurants and other enterprises selling foods and beverages in Denmark we diminished the data to include observations engaged in the retail of foods (i.e. “DD”) in the Capital region of Denmark. Thus, we included observations by postal codes and with the following DB07 branch codes; “DD.10.71.20 – bakeries etc.”; “DD.47.10.99 – Food stores”; “DD.47.20.99 –” ; “DD.47.22.00 –Butcheries” ; “DD.47.22.00 –Fish retailers” ; “DD.56.10.99 – Restaurants, cantinas etc.”; “DD.56.30.99 – catering businesses” [14]. The first four digits correspond to the EU’s industry classification NACE rev. 1. 1 [15], whereas the last two digits are Danish subgroupings corresponding to the UN’s industrial classification ISIC.

### Classification of food outlets

We applied the following most commonly used food outlet classifications in the literature according to Wilkins *et. Al* 2019 [16]; fast food, restaurants, convenience stores, supermarkets, fruit and vegetable stores and miscellaneous (Table 1). As mentioned, the great variation in outlet classification has caused a heterogeneity in studies of the food scape and potentially blurred conclusions drawn from these. Thus, in order to ease future comparability, we examined the most commonly applied definitions according to Wilkins *et. Al* 2019 [17]. Consequently, we divided each food outlet classification into the following three definitions; narrow, moderate and broad (Table 1).

Branch codes from the Smiley Register are too broadly defined to distinguish between different types of food outlets. Therefore, to automatically assign food outlets to a classification we applied a systematically name-based recognition procedure searching through the Smiley Register combining branch codes with a prespecified name or word describing “commonly known” food outlet types or common products from specific types of food outlets. Examples could be “McDonalds” or “burger” combined with a registered branch code “DD.56.10.99” being classified as fast food in this study; “7-eleven” combined with branch code “DD.47.10.99” classified as a convenience store; “Noma” combined with branch code “DD.56.10.99” classified as a restaurant. Often, the registered names are at best approximations of the actual name of the food outlet i.e. “banner names” thus leaving several food outlets unclassified from this systematic procedure. Therefore, we performed a subsequent manual virtual audit procedure on these. This procedure classified or excluded the remaining data based on predetermined criteria (Additional file 2) assessing information on both the internal and external appearance of the food outlet. Outlet names and addresses were applied in Google and/or Google street view to provide the information needed for classification (e.g. addresses with a photograph, retailer websites or consumer reviews). This procedure has been referred to as “Google-truthing” by Cohen et al . [18] and is a virtual form of ground-truthing streets. Consequently, each food outlet in the Smiley Register was classified from predetermined criteria using a combination of i) branch code, (ii) outlet name, (iii) Google Street View (GSV) images and (iv) other information available online (Additional file 2). All food outlets inaccessible to the greater public (e.g. located in hospitals, amusement parks, hotels etc.), irrelevant for the present study (e.g. selling mainly nature/health products, alcohol, candy and other specialties) or kitchens handling food for private purposes (e.g. work canteens, nursery homes etc.) were discarded. Duplicates were identified by name and address, checked manually, and eliminated for each of the downloaded Smiley datasets.

### Ground-truthing

During ground-truthing, both sides of all main streets within the selected areas were audited from September 2020 to May 2021. Surveyors were students trained in administering the survey apps through pilot testing and by using a standardized protocol developed for the purpose. Using the “ArcGIS survey123” app installed on a smartphone, location (i.e. geocoordinates as decimal degrees, preferably with a ± 5 m spatial accuracy when possible) and classification (i.e. the food outlet type) were assessed at the entrance of each identified food outlet. Completed responses were submitted directly to an ArcGIS account on “Survey123 online”. The survey was created in “Survey123 Collector” and structured as a filtered questionnaire (Additional file 3) based on similar criteria applied to manually classify food outlets from the Smiley Register (Additional file 2). Thus, by completing the survey, each food outlet was geographically located and automatically classified into type based on the combination of answers. Subsequently, classifications were divided into narrow, moderate, and broad definitions. Food outlets that appeared to be permanently closed were registered by name and location, but not classified. As ground-truthing was GPS assisted, a web map (Additional file 1) was developed in “ArcGIS online” for the surveyors to navigate within the boundaries of each grid. “Collector for ArcGIS” app was used for displaying the map of the streets in study grids while locating the surveyor by GPS. Each audited food outlet was displayed in the map, thus keeping track of records submitted while in the field. Surveyors were blinded to data from the Smiley Register. The time aspect of going through a study area varied depending on how densely populated the grid was, thus indirectly reflecting the number of food outlets present. A densely populated grid containing e.g. 15 food outlets and audited by one observer could take 20 minutes while a dispersedly populated grid containing e.g. 5 observations could take 5 minutes (excluding transport to and from grids).

### Food retailer matching process

Ground-truthed data were downloaded from “Survey123 online” and mapped in ArcGIS Pro together with data from the Smiley Register. For each day of ground-truthing, food outlets were visualized together in the same map. Subsequently, outlets were assessed by the same rater from a matching approach considering outlet name and location combined. Such a combination is relevant in study designs that apply outlet names from the secondary data source to extract different types of food outlets [19]. A match was made if names of two food outlets within a study grid cell were considered either same, similar, or tolerable. Discrepancies in names were allowed due to the known discrepancies between registered names in the Smiley Register and actual “banner names” of the food outlet. A similar name-match could be “Ristorante da Claudio” registered in the Smiley Register as “Da Claudio”. A tolerable name match suggests a similar type of retailer and product line in both names e.g. “Dominos” registered as “Pizza Group”. If a match was found outside the study area it was allowed within a distance of 50 m from the other food outlet. This relatively short distance was set in the perspective of considering the Smiley Register in future research as an exact geographical representation of the food environment i.e. for determining exact measures of access such as proximity. Further, by considering food outlets primarily within the randomly selected grid we aimed to avoid an unintentional underestimation of validity measures. Thus, we needed this spatial tolerance criterion in order to capture the relevant food environment for the present study. Consequently, combing name and location, a considered match could be two food outlets having similar names (e.g. ‘Mon Solo’ registered as ‘Non Solo Trattoria’) at a similar location (i.e. within the study grid or < 50 m apart if one food outlet was registered outside the grid).

### Statistics

From the matching process we identified true positives (TP: food outlets identified by both the Smiley Register and from ground-truthing), false positives (FP: food outlets identified only by the Smiley Register data) and false negatives (FN: food outlets identified only from ground-truthing). We calculated the sensitivity and positive predictive value (PPV) to assess the validity of applying a systematic procedure combined with “google-truthing” to identify, locate and classify food outlets in the Smiley Register. Sensitivity (TP/(TP + FN)) calculate the capability of the Smiley Register to correctly capture food outlets that are actually present in the field (only food outlets identified from ground-truthing were considered). Thus, a high sensitivity indicates no excessive undercount in the Smiley register. PPV (TP/ TP + FP) indicate the proportion of listed food outlets in the Smiley Register that was also present in the field (only food outlets in the Smiley Register were considered). Thus, a high PPV indicates that food outlets listed in the Smiley Register were located and open where they were listed to be. For the true positives we further evaluated whether they were given the same classification from the field-survey during ground-truthing as given from the systematically name-based procedure combined with “google-truthing”. This was assessed from calculating PPV’s across each food outlet type and specified as PPV (95% CI). Food outlets that were identified during ground-truthing but found irrelevant for the present study according to Table 1 (e.g. ice cream outlets or outlets primarily serving drinks) were excluded from both datasets if they were true positives. We interpreted agreement measures according to the Landis scale (< 0.00 poor, 0.00–0.20 slight, 0.21–0.40 fair, 0.41–0.60 moderate, 0.61–0.80 substantial, and 0.81–1.00 almost perfect reliability) [20], which has been used to interpret positive predictive values and sensitivities in a systematic review and meta-analysis of the validity of commercially available business data [9]. Further, we determined the proportion of food outlets across type that was classified solely from the systematic name-based procedure. The distance between the geocoordinates given in the Smiley Register and those registered from ground-truthing was examined and positional errors were calculated as median ± interquartile range. Finally, from the 32 expected empty grid cells we determined the proportion of true empty grids that were correctly identified from the Smiley Register as not presenting food outlets. All spatial analyses were conducted in either QGIS 3.10.0 or ArcGIS Pro 2.5.2 online (ESRI, Redlands, California), all statistical analyses and systematic procedures were conducted in SAS 9.4 and all manual data management was performed in Microsoft Excel.

## Results

In the 361 grids, ground-truthing identified 1887 food outlets compared with 1861 identified in the Smiley Register. Of the 1887 food outlets identified in the field, 469 food outlets did not have a match in the Smiley Register (Table 2). Of the 1861 food outlets identified in the Smiley Register, 443 were not identified in the field. Thus, considering match on location (regardless of classification), the sensitivity and PPV were 0.75 and 0.76, respectively. The distance between the coordinates of food outlets given in the Smiley Register and those found during ground-truthing had an accuracy in location of 13.61 ± 14.23 m (median ± interquartile range).

**Table 2:** Two-by-two table for identification of food outlets in the Smiley register and from ground-truthing in the Capital region of Denmark 2020–2021

		Food outlets identified from ground-truthing		
		Present	Absent	Total
Food outlets identified in Smiley data	**Present**	1418	443	1861
	**Absent**	469	–	
	**Total**	1887		

Sensitivity 75%, Positive predictive value 76%

The total number of classified food outlets listed in the Smiley Register was 1831 (7 observations were reported insufficiently to apply a classification and definition, 23 observations were excluded as they were irrelevant). Of these,1388 were classified and defined similarly to ground-truthed data. Across classification and definition the PPVs varied with highest PPVs for the moderate and broad definitions of fast food, convenience stores and supermarkets (PPV ranging from 0.89–0.97) (Table 3). As an example, out of the 430 fast food outlets identified in the Smiley Register as being within the moderate fast food definition, 400 were classified the same from direct observation during ground-truthing (PPV (95% CI) =0.93(0.91–0.95)). Similarly, out of the 302 restaurants outlets identified in the Smiley Register as being within the narrow restaurant definition, 184 were classified the same from direct observation during ground-truthing (PPV (95% CI) =0.61(0.55–0.66)). Considering the proportion of food outlets classified solely from the systematic name-based procedure (Table 3), supermarkets and convenience stores were found to be classified the best with little need for manual classification: 96% of the supermarkets and 72% of the convenience stores within the moderate definition were classified from the systematic name-based procedure). In contrast, more than half of the restaurants were identified and classified manually following the systematic procedure.

**Table 3:** Positive predictive value (95% CI) and proportion of food outlets classified automatically across food outlet classification and definition

Food outlet classification	Definition	True positives ^a^	False positives	PPV (95% CI)	% classified from the systematic name-based procedure
**Restaurant**	Narrow	184	118	0.61 (0.55–0.66)	28
	Moderate	302	147	0.67 (0.63–0.72)	37
	Broad	402	138	0.74 (0.71–0.78)	45
**Fast food**	Narrow	13	6	0.68 (0.48–0.89)	68
	Moderate	400	30	0.93 (0.91–0.95)	59
	Broad	528	44	0.92 (0.90–0.95)	61
**Convenience store**	Narrow	71	12	0.86 (0.78–0.93)	63
	Moderate	108	14	0.89 (0.83–0.94)	72
	Broad	111	14	0.89 (0.83–0.94)	73
**Supermarket**	Narrow	48	9	0.84 (0.75–0.94)	84
	Moderate	167	5	0.97 (0.95–1.00)	96
	Broad	280	17	0.94 (0.92–0.97)	87
**Fruit and Vegetables**	–	17	22	0.44 (0.28–0.59)	–
**Miscellaneous**	–	108	5	0.96 (0.92–0.99)	81

*CI* Confidence interval, *PPV* Positive predictive value ^a^ Number of food outlets correctly located (1831) and number of outlets wrongly classified (443) but found to be in the correct location

Of the 32 grid cells expected not to include food outlets, 31 were correctly identified as “empty grid cells” (97%). The one observation found in an expected empty grid was a hotdog stand placed in an industrial area. These often have the owner’s residential address registered in the Smiley register but are located at central sites during the day.

## Discussion

According to the Landis scale the sensitivity and positive predictive value of the Smiley Register would be considered as substantially reliable. Hence, 75% of food outlets observed during ground-truthing had a match on location and outlet name in the Smiley Register, while the probability that food outlets listed in Smiley Register were located and open where they were listed to be was 76%.

The strength of agreement across classifications from ground-truthing and the systematically name-based procedure was considered substantial to almost perfect. For food outlets that were correctly identified and located, the systematic and manual procedure classified the outlets almost perfect across definition for convenience stores, fast food outlets, and supermarkets (PPV = 0.86–0.89, 0.68–0.93 and 0.84–0.97, respectively) and substantially for restaurants (PPV = 0.61–0.74). The fruit and vegetable classification were characterized by a small sample size (*N* = 22) and a moderate PPV (0.44). As is seen from Additional file 2, fruit and vegetable outlets do not have their own branch code like other specialty food outlets (e.g. butcheries, fish retailers and bakeries). Initially we did not include fruit and vegetable outlets as a separate classification, thus no relevant names were included to identify these from the name-recognition procedure. During the manual processing of data from the Smiley Register we realized that non-chain convenience stores initially defined as “minimarkets” in this study also had a great variety of fruit and vegetables. Thus, we included those food outlets in a separate classification named “fruit and vegetables”. However, considering the accompanying sample size and PPV this classification needs improvement for example by considering specific names of “commonly known” fruit and vegetables outlets or common products from these outlets.

When contrasting narrow to moderate and broad definitions across all classifications, the PPVs where generally highest for the moderate and broad definitions. This was partly expected as the narrow definitions generally comprise large food outlet chains and thus omit all non-chains, that often provide very similar food and thus would apply under similar classification criteria. A disadvantage that follows from dividing each classification into such definitions is the small sample size, especially for the narrow definition. Theoretically, the moderate definition would capture food outlets with a more consistent type of food provision compared with the broad definition. Further, the moderate definition has been most commonly applied across food outlet types in the foodscape literature [16]. Thus, to enable comparability in future studies and considering that the moderate and broad definitions had similar accuracies in this study, applying the moderate definitions to food outlets of the Smiley register would be preferable.

A moderate to substantial accuracy of secondary food outlet data is commonly found in similar validation studies. Lebel et al. [9] evaluated 20 studies from 2006 to 2015 conducted in the United Kingdom, the United States, Canada and Denmark. All studies validated at least one secondary data source against a primary data source, either ground truthing or government lists (i.e. food establishment inspections or licensing records). Across all food outlet subsamples, the median PPV was 77% (Interquartile range = 30%) and median sensitivity 60% (Interquartile range = 37%). However, the PPV ranged from 38 to 95% while the sensitivity ranged from 40 to 98%. Notably, great variations are found across study designs. As an example, all of the examined studies by Lebel et al. [9] are based on commercial data and not administrative data as in this study. Further, not all are validated against the gold standard of ground truthing. Other methodological choices that varies include study area (e.g. country, region, rural, urban, mixed) and matching criteria.

Matching criteria are sometimes described as being “strict”, requiring a match based on outlet name or “relaxed”, requiring a match between the type of food outlet/classification and street names [19, 21]. Another possibility is “location matching”, requiring a match on excact street name and house number. In the present study we applied matching criteria that can be considered as strict, but a great variety in matching criteria is found across studies. Consequently, methodological choices influence agreement statistics and their comparability across other apparently similar studies. Agreement statistics are also found to vary but are relatively high in studies published after 2015. Caspi and Friebur [22] and Wong et al. [23] applied similar matching criteria as to our study for comparison of commercial data against ground-truthed data. Caspi and Friebur (2016) found an average PPV of 0.57 and a sensitivity of 0.62 across three town/rural areas in Minnesota, US. Similarly, Wong et al. [23] found moderate PPVs in two different data sources: 0.58 for the academic-government partnership data and 0.46 for commercial data. Sensitivities were higher and more similar for both data sources: 0.90 and 0.93, respectively. Other studies have applied more than one matching approach in the comparison of different sources of secondary food outlet data and ground-truthed data. As in this study, Díez et al. [24] applied location/name matching but also location matching alone to examine administrative food retailer data from Madrid, Spain. The first matching procedure resulted in substantially lower agreement statistics then the latter, with a sensitivity of 0.55 (CI:0.44–0.64) and a PPV of 0.45 (CI:0.37–0.54) compared to a sensitivity of 0.95 (CI:0.89–0.98) and a PPV of 0.79 (CI:0.70–0.85). Wilkins et al . [19] examined both commercial and administrative data from urban and rural areas in Leeds, England. A relaxed matching approach resulted in high PPVs for both data sources, though highest were attained for the administrative data (0.91, CI: 0.89–0.93) vs. the commercially data (0.86, CI: 0.84–0.88). The sensitivity was similar in both datasources; 0.84, CI: 0.82–0.86 vs. 0.81, CI: 0.78–0.83, respectively. When strict matching criteria were applied, the strength of agreement decreased for both the administrative data (PPV:0.87, CI: 0.85–0.89; sensitivity: 0.81, CI: 0.78–0.83) and the commercially data (PPV: 0.79, CI: 0.77–0.82; sensitivity: 0.74, CI: 0.72–0.77). Finally, in a Dutch study, Canalia et al. [25] evaluated commercial data and found a sensitivity of 0.91 and a PPV of 0.90 from location matching. With a relaxed matching approach the sensitivity and PPV were 0.97 and 0.85, repectively. Notably, for studies using matching criteria based on both name and location, PPV’s were generally lower and more similar to results from this study than for those applying relaxed matching criteria or location matching alone. Because we aimed to examine the validity of applying a combined systematic and manual procedure for classifying food outlets, we were not able to consider a match based on location and classification as the latter information was blinded during the matching process. Given the high density of food outlets in some study areas a match could not rely on location matching alone. Thus, we considered location along with other information to correctly match closely located food outlets. In this aspect, we found outlet name to be the best of possible choices; branch code being another alternative but being too broad.

Food outlets found in the field but not in the Smiley Register (false negatives) may result from i) poor registration; ii) unauthorized food outlets and iii) chosen matching criteria (e.g. difference in food outlet names may have prevented a potentially correct match). Disregarding the false negatives, we found that 76% of the food outlets listed in the Smiley Register were located and open where they were listed to be. This is considered a conservative estimate as the number false positives may have been influenced by the three following issues; i) we did not track the route of each surveyor. Surveyors may have missed some mains streets where food outlets were located and further, some food outlets may have been located away from main streets; ii) though the Smiley Register is updated daily the inspection frequency of food outlets is typically only one to four times a year depending on risk evaluation. Consequently, food outlets with a low inspection frequency may have closed permanently or changed name in the timespan between inspection and ground-truthing, thus potentially overestimating the number of false positives. On March 13, 2020 Denmark imposed a strict lockdown due to the COVID-19 pandemic. Consequently, restaurants and take-away food outlets were allowed serving only take-away. This enforced some food outlets to close immediately, while lack in sales the following year during a gradual reopening caused temporary or permanent shutdowns. Within this period field data were gathered for the present study. Examining the inspection dates registered in the Smiley Register we found that 51% of the false positive food outlets were last inspected before March 13, 2020 while this was the case for 41% of the true positives (results not shown). Thus, the number of false positives were similar before and after lockdown while more true positives had an inspection date during or after the lock down than before. A third issue potentially influencing the number of false positives arise from our study design. We experienced that food outlets in shopping malls were registered in the Smiley Register as located by the main entrance of the mall and not the entrance of the food outlet as in this study. This would potentially cause a lack of match due to our spatial tolerance criterion of 50 m. Another problem that follows from the study design occurs when only the main entrance of the shopping mall is a part of the studied grid and the rest of the mall is not. Consequently, the issues listed above could contribute to a higher number of false positives that in turn would underestimate the PPV of the total sample.

Our results show that if a food outlet is listed in the Smiley Register, it likely exists within a distance of 13.61 ± 14.23 m (median ± interquartile range) from the registered location. Yet, applying the developed procedure to the Smiley Register likely only captures a fraction of the food outlets and thus does not reflect an exact copy of the foodscape. This may have no greater impact if one wants to generate spatial measures of the foodscape based on this register. If such a measure is described in terms of density within a specific area (e.g. number of food outlets within grids, residential census tracts, home-centered buffers or kernel-density estimates) or in relative terms (e.g. proportion of fast food outlets out of total) then the “missing” fraction of food outlets in the Smiley Register is not necessarily important as long as it is not skewed in regards to food outlet type. Considering a density measure, 10 unmatched food outlets could represent 5 false negatives and 5 false positives within an area. As long as they are within the same classification the expected impact from the foodscape would be the same as they would outweigh each other. Thus, seeing the Smiley Register as an alternative to researcher ground-truthed data can be a meaningful choice depending on the research objective. Regardless, acknowledging the accuracy and completeness of the Smiley Register is crucial if applied to describe and measure the foodscape.

Strengths of using this register for future research of the foodscape is the administrative nature and national coverage; All food outlets in Denmark are responsible for complying with the food regulations and thus need to register to be inspected and to be in business. Further, it is freely available and updated daily, which is a major asset given that the retail food environment is highly dynamic. By applying a systematic and manual procedure to the Smiley Register the data-gathering-process is less time consuming than ground-truthing, especially for those food outlet types with high a PPV that can be classified mostly from the systematic procedure (i.e. supermarkets and convenience stores). Denmark is geographically divided into five regions [26]. The geographical area of interest in the present study involved the Capital region (excluding the island of Bornholm). This region covers areas of high and low population density, has a great large diversity in sociodemographic characteristics and had a total population of 1,814,296 in year 2020 [27]. Given the coverage of both urban and rural areas and areas of diverse sociodemographic characteristics, the number and variety in the type of food outlets per study area is expected to be generalizable to other regions in Denmark. Provided that the quality of the Smiley Register is the same one would also be able to look back in time and consider temporary changes.

## Conclusion

In this study we evaluated the validity of an administrative data source (the Smiley Register) containing geographical locations and other information available to classify the type of food outlets against field audit data. By applying a systematic and manual procedure to the Smiley Register it was possible to identify, locate and classify food outlets with a substantial to almost perfect accuracy according to the Landis scale. Thus, information from the Smiley Register is considered to be representative to the Danish foodscape.

## Supplementary Information

**Additional file 1.** Map displaying the Capital region of Denmark (excluding the island Bornholm) (within dotted lines) illustrating the geographical distribution of the randomly selected grid cells (black boxes) for the ground-truthing. Each cell is 250x250m and contain at least one type of food outlet. 336 grids were selected; of these 3 were mistakenly placed outside the Capital region, while 4 grids were placed at an amusement park (i.e. not accessible to the greater public). These were discarded leaving 329 grids. Additionally, 32 grids were selected as being “empty” according the Smiley Register 2021. Map created in ArcGIS PRO.

**Additional file 2.** List of search terms and characteristics for identification, location and classification of food outlets in the Smiley Register; Search terms are mainly given for the moderate definitions. Further, search terms are given for coffee shops that are included in the broad definitions of restaurants and fast food.

**Additional file 3. **The classification tool behind the field survey applied during ground-truthing; By completing the survey, each food outlet is geographically located and automatically classified into type (white boxes) based on the combination of answers. The white boxes comprise the five most common food outlets classifications used in the literature i.e. fast food, restaurants, convenience stores, supermarkets, fruit and vegetable stores [16]. Light grey boxes supply information needed for the subsequent partitioning of each classification into three definitions; narrow, moderate and broad with inspiration from Wilkins *et. al* (2019a).

## Data Availability

The Smiley data and ground-truthing data that support these findings are available from the corresponding author upon reasonable request. Updated Smiley data are freely available through the Danish government inspection website, http://www.findsmiley.dk/english/Pages/About.aspx
