# Supplementary figures and images for: Validation of retail food outlet data from a Danish government inspection database
Source: Nutr J. 2022 Sep 27;21:60. doi: 10.1186/s12937-022-00809-6 (PMC9513017; doi:10.1186/s12937-022-00809-6)

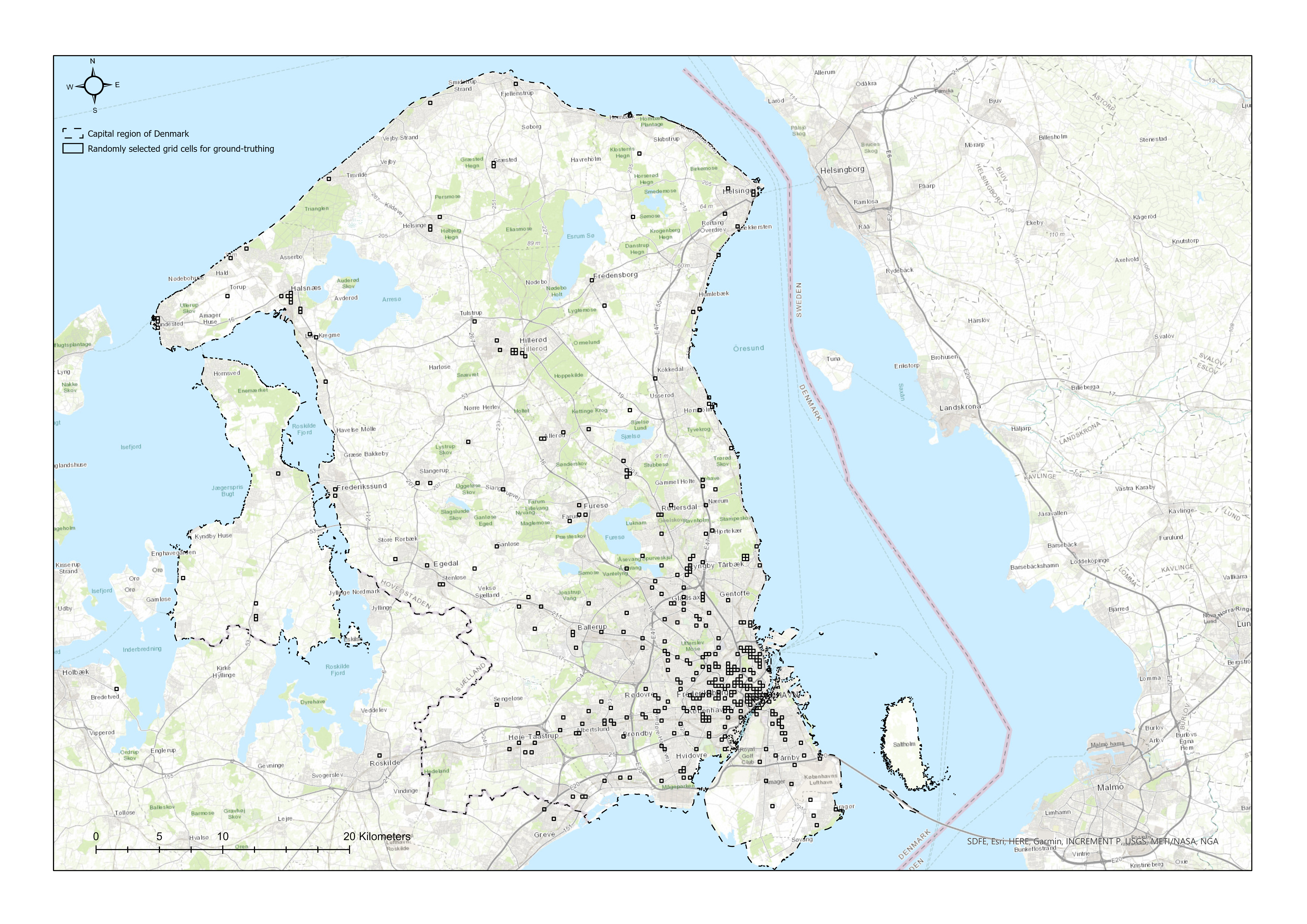

Supplement: Supplementary file 1 — Additional file 1. Map displaying the Capital region of Denmark (excluding the island Bornholm) (within dotted lines) illustrating the geographical distribution of the randomly selected grid cells (black boxes) for the ground-truthing. Each cell is 250x250m and contain at least one type of food outlet. 336 grids were selected; of these 3 were mistakenly placed outside the Capital region, while 4 grids were placed at an amusement park (i.e. not accessible to the greater public). These were discarded leaving 329 grids. Additionally, 32 grids were selected as being “empty” according the Smiley Register 2021. Map created in ArcGIS PRO. [file 12937_2022_809_MOESM1_ESM.jpg]

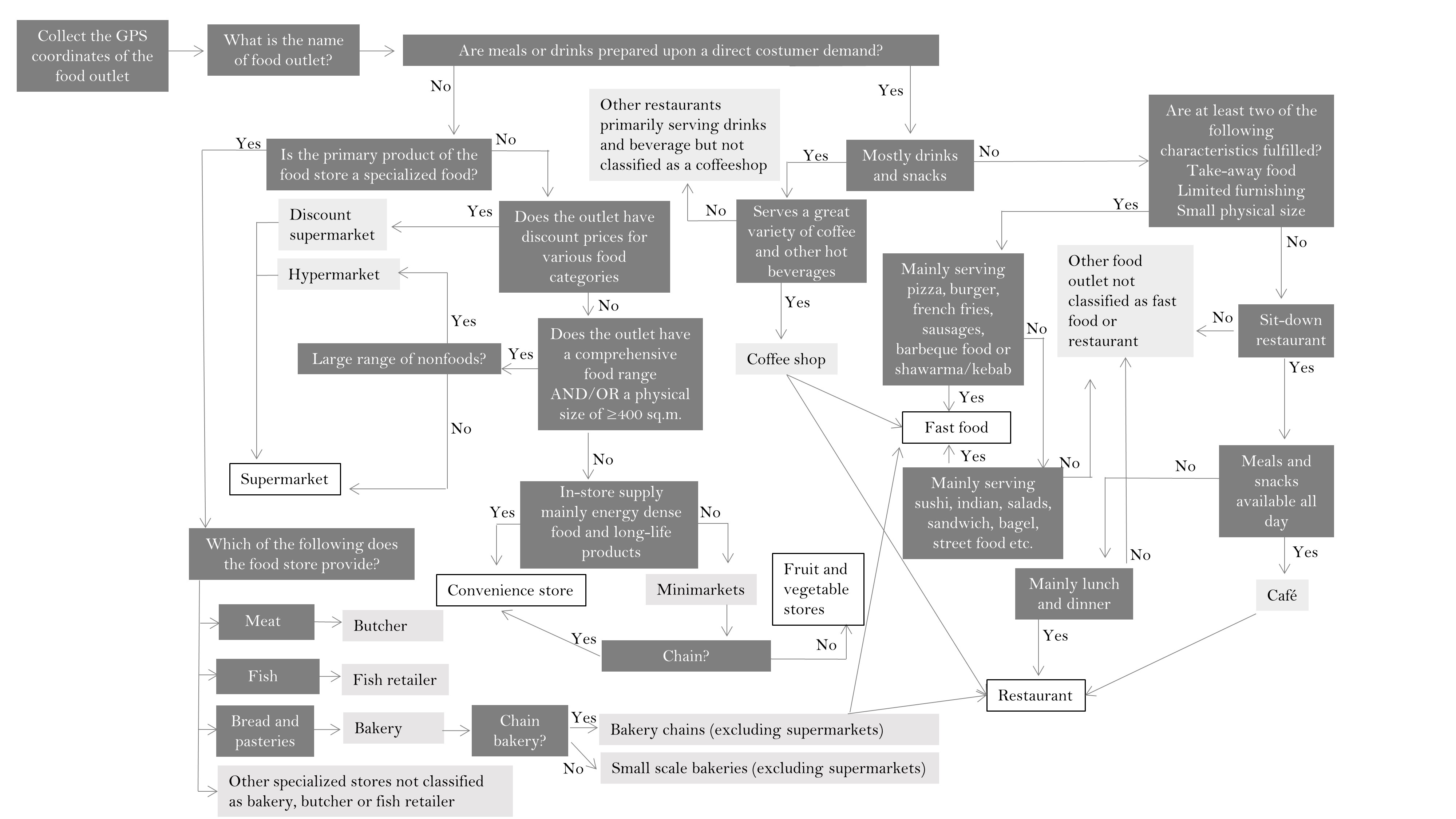

Supplement: Supplementary file 3 — Additional file 3. The classification tool behind the field survey applied during ground-truthing; By completing the survey, each food outlet is geographically located and automatically classified into type (white boxes) based on the combination of answers. The white boxes comprise the five most common food outlets classifications used in the literature i.e. fast food, restaurants, convenience stores, supermarkets, fruit and vegetable stores [16]. Light grey boxes supply information needed for the subsequent partitioning of each classification into three definitions; narrow, moderate and broad with inspiration from Wilkins et. al (2019a). [file 12937_2022_809_MOESM3_ESM.jpg]
