# Supplementary material for: Validation of retail food outlet data from a Danish government inspection database
Source: Nutr J. 2022 Sep 27;21:60. doi: 10.1186/s12937-022-00809-6 (PMC9513017; doi:10.1186/s12937-022-00809-6)
Supplement: Supplementary file 2 — Additional file 2. List of search terms and characteristics for identification, location and classification of food outlets in the Smiley Register; Search terms are mainly given for the moderate definitions. Further, search terms are given for coffee shops that are included in the broad definitions of restaurants and fast food. [file 12937_2022_809_MOESM2_ESM.docx]

| **Outlet type (**definition**)** | | **Inclusion criteria for the systematic name-based recognition procedure** | **Characteristics for manual examination in Google and for field validation** |
| --- | --- | --- | --- |
| **Fast food** (moderate)  This classification includes so-called pizzerias, burger joints etc. as defined from the Danish trade association and employer’s organization for the restaurant, hotel and tourism industry in Denmark (HORESTA) but also other fast food outlets serving fast food that’s is not necessarily high in energy, fats, and salt/sodium as pizza. | | Outlets within branch code DD.56.10.99 of the Smiley Register and with one of following words incorporated in outlet name registered in the Smiley Register: pizza, grill, pølse, hotdog, kebab, shawarma, falafel, burger, den sorte gryde, mcdonald, sunset, burger king, kentucky fried chicken, dürüm, steffs place, max, subway, aspendos, gastronomia, bella italia, domino, french chicken, king chicken, bagel, the bagel co, the bagel store, salat, sandwich, street food, street market, madbod, smørrebrød, wedofood, the bagel co, royal bagel, royal food, nordic noodle, nadias sandwich, dagens måltid. | Physically small outlets serving fast food primarily as take-away, with a small service area (<30sq.m), limited furnishing, counter service/no possibility of table reservation. |
| **Convenience** (broad)  This classification includes so-called convenience stores and minimarkets as defined from HORESTA | | Outlets within branch code DD.47.10.99 of the Smiley Register and with one of following words incorporated in outlet name registered in the Smiley Register: elite købmand, letkøb, circle k, uno x, ok, q8, shell, 7-eleven, shop’n play, nærkøb, dagligbrugsen, lokalbrugsen, spar, kwikspar, brugsen, min købmand, købmand, kiosk. | These outlets are typically gas stations or chain and grocery outlets. They are physically small compared with supermarkets (i.e. <400 sq.m.) typically with long opening hours (e.g. 24 hours or closing times later than supermarkets i.e. closes at 22 PM or later), with limited supplies compared with supermarkets and an in-store supply sparse of healthy and fresh foods such as fruits and vegetables, mostly consisting of energy dense food and long- life products. |
| **Supermarket** (moderate)  This classification includes so-called hypermarkets, supermarkets and discount supermarkets as defined from HORESTA | | Outlets within branch code DD.47.10.99 of the Smiley Register and with one of following words incorporated in outlet name registered in the Smiley Register: bilka, kvickly, superbrugsen, irma, føtex, superbest, superspar, eurospar, løvbjerg, abc lavpris, meny, fakta, netto, kiwi, rema1000, aldi, lidl. | These outlets are typically physically large outlets (>400 sq.m) with a wide range of foods.  Hypermarkets typically also have a comprehensive range of non-foods and items such as clothing and electronics while supermarkets and discount supermarkets have a smaller range of non-foods. Discount supermarkets have discount prices compared with supermarkets and hypermarkets. |
| **Restaurants** (moderate)  This classification includes restaurants and cafés as defined from HORESTA | | Outlets within branch code DD.56.10.99 in the Smiley Register with the following specific names or words: bone, chicago roasthouse, flammen, hereford beefstouw, jensens bøfhus, mash, halifax, cocks & cows, the burger, madklubben, hanzo, alabama social, food club, cofoco, scarpetta, høst, vækst, les trois cochons, oysters, vespa, spuntino, the italian, jah izakaya & sake bar, llama, italy søborg, geranium, aoc, kadeau copehagen, era ora, formel b, kokkeriet, studio at the standard, søllerød kro, 108, relæ, marchal, kong hans kælder, the south indian, sushimania, sticks n sushi, punjab, magasasa, nimb, lêlê, kiin kiin, letz sushi, iki sushi, jagger, highway 66, gorm*s, armandos, almanak, applaus, altopalato, orangeriet, arti`kok, asia house, asian palace, atelier 85, atlas bar, avanti, azzurra, fiskeværksted, bakkarøgeriet, ban phai, nordic noodles, izumi, ribhouse, sachi sushi, restaurant, restauranten, restauration, rest., ristorante, restorante, restaurant, trattoria, kro, hotel, steak, india, gourmet. Outlets within branch code DD.56.10.99 or DD.56.30.99 in the Smiley Register with the following specific names or words: ikea, dalle valle, palæo, atelier september, wulff & konstali, cafe, café, cafè, cafeen, caféen, bistro, grannys house, retreat. | These outlets are sit-down restaurants providing waited table service serving either primarily lunch and evening meals or readily prepared meals, buffet and snacks all day. |
| **Coffee shops**  This classification is not applied in itself in the present paper. It is applied in the broad definition of restaurants and convenience stores respectively. | | Outlets within branch code DD.56.10.99 in the Smiley Register with the following specific names or words: ricco, starbucks, shabaz, baresso, original coffee, espresso house, resso roasthouse, the coffee collective, kaffe, kaffebar. | These outlets include both larger chains and small outlets mainly serve hot beverages and snacks with a limited selection of ready-made foods such as sandwiches, pastry etc. |
| **Fruit and Vegetable stores** | | Outlets within branch code DD.47.10.99 of the Smiley Register.  No name-recognition procedure as these were defined later in the process from the food outlets initially classified as “minimarkets” I then further divided into either convenience stores or fruit and vegetable stores (Additional file 3) | These outlets are typically non-chain grocery outlets (excluding farm shops) primarily retailing fruits and vegetables and typically also long-life products but with a limited supply compared with supermarkets. Outlets are physically small compared with supermarkets (i.e. <400 sq.m.) typically with long opening hours (e.g. 24 hours or closing times later than supermarkets i.e. closes at 22 PM or later).  (These outlets are those outlets that are not grocery or convenience chains from the Convenience type). |
| **Miscellaneous** | Bakeries | Food outlets within branch code DD.10.71.20 and DD.47.10.99 of the Smiley Register with the following specific names or words: bakery, bager, konditor, brød, bageriet, bakery by hermann, bodenhoffs bageri, byens bager, city bakery aps, emmerys, farumhus a/s, guldbageren, holms bager, lagkagehuset, meyers bageri, reinh van hauen. | Small-scale bakery outlets and chain outlets with a selection of bread and pastry as main product (excluding bakeries in supermarkets). |
|  | Butcher | Food outlets within branch code DD.47.22.00 of the Smiley Register. | Small-scale food stores with a selection of meat as main product (not as part of a supermarket). |
|  | Fish Retailer | Food outlets within branch code DD.47.23.00 of the Smiley Register. | Small-scale food stores with a selection of fish as main product (not as part of a supermarket). |
